# Eosinophils Increase Neuron Branching in Human and Murine Skin and *In Vitro*

**DOI:** 10.1371/journal.pone.0022029

**Published:** 2011-07-21

**Authors:** Erin L. Foster, Eric L. Simpson, Lorna J. Fredrikson, James J. Lee, Nancy A. Lee, Allison D. Fryer, David B. Jacoby

**Affiliations:** 1 Department of Molecular Microbiology and Immunology, Oregon Health & Science University, Portland, Oregon, United States of America; 2 Department of Dermatology, Oregon Health & Science University, Portland, Oregon, United States of America; 3 Department of Biochemistry, Mayo Clinic, Scottsdale, Arizona, United States of America; 4 Division of Pulmonary and Critical Care, Department of Medicine, Oregon Health & Science University, Portland, Oregon, United States of America; Beth Israel Deaconess Medical Center, United States of America

## Abstract

Cutaneous nerves are increased in atopic dermatitis, and itch is a prominent symptom. We studied the functional interactions between eosinophils and nerves in human and mouse skin and in culture. We demonstrated that human atopic dermatitis skin has eosinophil granule proteins present in the same region as increased nerves. Transgenic mice in which interleukin-5 (IL-5) expression is driven by a keratin-14 (K14) promoter had many eosinophils in the epidermis, and the number of nerves was also significantly increased in the epidermis. In co-cultures, eosinophils dramatically increased branching of sensory neurons isolated from the dorsal root ganglia (DRG) of mice. This effect did not occur in DRG neurons co-cultured with mast cells or with dead eosinophils. Physical contact of the eosinophils with the neurons was not required, and the effect was not blocked by an antibody to nerve growth factor. DRG neurons express eotaxin-1, ICAM-1 and VCAM-1, which may be important in the recruitment, binding, and activation of eosinophils in the region of cutaneous nerves. These data indicate a pathophysiological role for eosinophils in cutaneous nerve growth in atopic dermatitis, and suggest they may present a therapeutic target in atopic dermatitis and other eosinophilic skin conditions with neuronal symptoms such as itch.

## Introduction

Atopic dermatitis is characterized by itch, which greatly affects the quality of life of patients [Jacquet, 1904 and [1]]. The itch often begins before any lesions appear, and marks on the skin can be limited to excoriations, or scratches, made by the patient. Patients with atopic dermatitis experience itch instead of pain when tested with mechanical, electrical, low pH, or heat stimuli [2]. The sensory neurons that transmit itch are primary afferents whose cell bodies are in the dorsal root ganglia (DRG). These free nerve endings in the epidermis and upper dermis can be activated by a variety of stimuli, including proteases, neurotrophins, cytokines and other small molecules (reviewed in [3]).

The mechanisms for enhanced itch sensations in atopic dermatitis are unclear. One potential mechanism is an increase in nerve endings in atopic dermatitis skin [4]. Specifically, there are more nerve fibers in the papillary and upper dermis as disease progresses from clinically normal-appearing, or *non-lesional*, skin to active disease, or *lesional*, skin [5].

Eosinophils have been linked to atopic dermatitis for well over forty years, due to high numbers circulating in the blood of atopic dermatitis patients [6]. Serum concentrations of eosinophil granule proteins correlate with the severity of atopic dermatitis [7], [8], and peripheral blood eosinophils from patients have more neurotrophin receptors and more functional activity in response to neurotrophins than eosinophils from healthy controls [9]. Intact eosinophils are not found in high numbers in atopic dermatitis biopsies, leading some to question whether these cells have a role in the pathogenesis of this disease. However, the presence of eosinophil granule proteins in lesional skin suggests that activated eosinophil are present, but not identifiable, having degranulated [10], [11].

Previously, we showed that eosinophils interact with nerves in the airways of patients with asthma, in animal models of asthma, and in culture. Eosinophils are found adjacent to nerves in airway biopsies of humans who died from asthma attacks, a histological finding that is recapitulated in antigen-challenged guinea pigs and rats [12]. Primary cultures of parasympathetic neurons from guinea pig and human airways express eotaxin-1, as well as ICAM-1 and VCAM-1, and these participate in binding of eosinophils to neurons [13], [14]. The association of eosinophils with airway nerves is important in the pathophysiological changes that lead to airway hyperreactivity [12], [15].

Eosinophils communicate with other cell types through a variety of specific signals. They can act as antigen-presenting cells, release cytokines and chemokines after activation, or release granule proteins, such as major basic protein (MBP) and eosinophil peroxidase (EPO) [16], [17]. They also constitutively synthesize specific neurotrophins, including nerve growth factor (NGF), brain-derived neurotrophic factor (BDNF), and neurotrophin-3 (NT-3), and they can be triggered to release these factors upon stimulation [9], [18].

In this study, we investigated the physical and functional relationship between eosinophils and sensory nerves in the skin of humans with atopic dermatitis, in a mouse model of atopic dermatitis, and in co-cultures of eosinophils with primary sensory neurons. We report here that eosinophil granules are located near neurons in the upper, or papillary, dermis of human skin, the region in which nerves are also increased. In a transgenic mouse in which IL-5 is driven by a K14 promoter, eosinophils localize to the epidermis and lead to increased nerves in this region. In culture, eosinophils directly cause branching of sensory neurons, through a mechanism that does not involve NGF. These data indicate a potential mechanism and therapeutic target for the increase in nerves seen in human atopic dermatitis.

## Results

### Human skin biopsies are representative of healthy skin and different stages of atopic dermatitis

**Figure 1:**
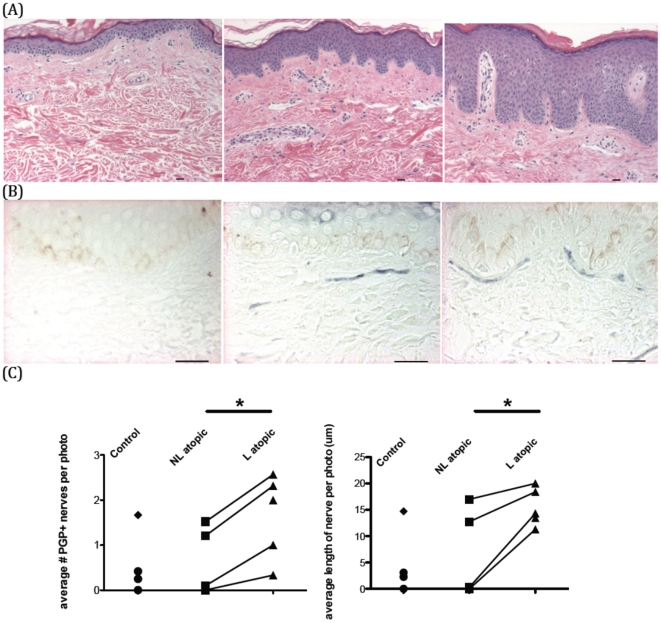
Atopic dermatitis lesional skin has more nerves than paired non-lesional skin. (A) Human skin biopsies are typical of healthy control skin (left), non-lesional atopic dermatitis (center), and lesional atopic dermatitis (right). (B) Nerves were stained (in gray), and representative sections are shown of healthy control (left), non-lesional (center) and lesional atopic dermatitis (right). (C) Quantification of number and length of nerves in healthy control or atopic dermatitis skin. Paired t-test was performed between lesional and non-lesional skin biopsies from the same subject. Outlier control subject with high blood eosinophils and increased nerves is represented by diamond. * denotes significantly different from control. Scale bar in photographs = 50 um.

Skin biopsies were gathered from five healthy volunteers and six subjects with atopic dermatitis. Patients with atopic dermatitis had not used topical corticosteroids for a minimum of a week before the biopsies were taken. Each subject was examined by a dermatologist and confirmed to have atopic dermatitis, both by clinical exam and patient and family history. All atopic dermatitis subjects were previously diagnosed with the Hanifin and Rajka criteria [1]. One biopsy was taken from each healthy control, and two were taken from each atopic dermatitis subject, for paired lesional (L) and non-lesional (NL) skin. Non-lesional skin throughout this report refers to normal appearing skin from an atopic dermatitis patient. Subject characteristics and biopsy sites are shown in Table 1. All patients had positive family and personal history for atopic dermatitis, including allergies and asthma. Hematoxylin and eosin (H&E) staining of the human skin biopsies confirmed the clinical diagnoses of healthy control, non-lesional atopic dermatitis or lesional atopic dermatitis (Figure 1A).

**Table 1:** Human skin biopsies.

Subject	Skin type	Samples taken	Region
001	Atopic Dermatitis	Lesional/Non-lesional	Flexor knee
002	Healthy Control	Normal	Interior arm
003	Healthy Control	Normal	Interior arm
004	Atopic Dermatitis	Lesional/Non-lesional	Flexor knee
005	Atopic Dermatitis	Lesional/Non-lesional	Flexor elbow
006	Atopic Dermatitis	Lesional/Non-lesional	Exterior arm
007	Healthy Control	Normal	Interior arm
008	Healthy Control	Normal	Interior arm
009	Healthy Control	Normal	Interior arm
010	Atopic Dermatitis	Lesional/Non-lesional	Interior arm
011	Atopic Dermatitis	Lesional/Non-lesional	Extensor elbow

**Table 2:** Eosinophil peroxidase (EPO) staining in human skin biopsies.

Name of sample	avg # EPO+ clusters	EPO+ regions of skin
**NORMAL:**		
002 normal	1.5	papillary dermis
003 normal	0.6	basement membrane zone
008 normal	0.25	papillary dermis
009 normal	1	reticular dermis
**NON-LESIONAL**		
001 non-lesional	1.8	papillary dermis
004 non-lesional	2.43	papillary dermis
005 non-lesional	2.36	papillary dermis
006 non-lesional	3	papillary+reticular dermis
011 non-lesional	1.833	papillary dermis
**LESIONAL**		
001 lesional	2.5	papillary+reticular dermis
004 lesional	5.08	papillary+reticular dermis
005 lesional	2.3	papillary+reticular dermis
006 lesional	3.125	papillary+reticular dermis
010 lesional (unpaired)	4.36	papillary dermis
011 lesional	2.75	papillary dermis

### Atopic dermatitis lesional skin biopsies have more nerves than non-lesional skin from the same subjects

Biopsy sections were stained with an antibody to the pan-neuronal marker PGP9.5. Photographs were taken along the length of the skin section, and nerves staining positively for PGP9.5 were counted in the papillary dermis and basement membrane zone of the skin. Representative photomicrographs of normal, non-lesional and lesional skin are shown with quantification (Figure 1 B, C). Nerves were increased, both in number and in length, in lesional atopic dermatitis skin, compared to each non-lesional skin sample from the same patient. Overall, nerves were also increased compared to healthy control skin, except for one healthy control with many more cutaneous nerves. It may be significant that this outlier subject had high numbers of eosinophils in the blood and also had increased eosinophil granule proteins in the skin.

### Atopic dermatitis skin biopsies have more eosinophil granule proteins, which are located around nerves in the papillary dermis

**Figure 2:**
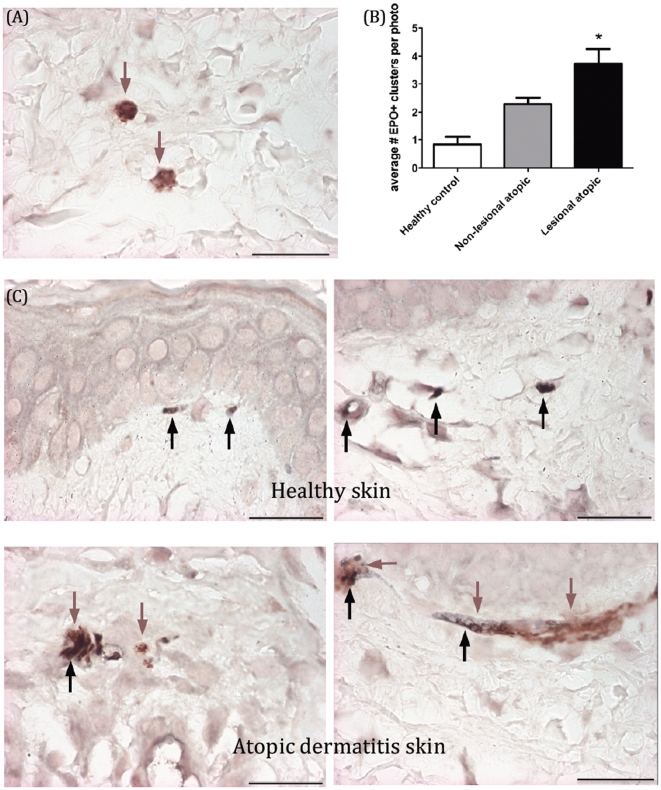
Eosinophil granule proteins are present in atopic dermatitis skin, in regions near nerve increases. A) Human skin biopsies were stained with anti-EPO antibody (dark red-brown). A representative photomicrograph is shown of lesional skin. B) Quantification of EPO-positive clusters in skin. C) Representative photomicrographs of double immunohistochemistry using anti-PGP9.5 (black) for nerves and anti-EPO (red-brown). Top panels are two samples of healthy control skin. Bottom panels are two lesional atopic dermatitis samples. Bottom left shows a nerve with EPO+ cells nearby, while bottom right shows a nerve overlaid with EPO. * denotes significantly different from control. Scale bar = 50 um.

In order to determine if eosinophil localization to nerves in the skin occurs in atopic dermatitis biopsies, we first performed single immunohistochemistry with an antibody to eosinophil peroxidase (EPO), a protein present in eosinophil granules. EPO was present in lesional atopic dermatitis skin at higher quantities than normal or non-lesional skin, and it was often located in the papillary dermis (Figure 2 A,B and Table 2). This localization correlated with both the location and stage of disease in which nerves are increased. Intact eosinophils were present in some lesional skin sections, more often in blood vessels (data not shown), suggesting that eosinophils that have migrated into tissues tended to degranulate. In addition, double immunohistochemistry for eosinophil peroxidase and PGP9.5 determined that eosinophil peroxidase could be found around nerves in the papillary dermis, especially in lesional skin samples (Figure 2C).

### Mast cells are increased in non-lesional atopic dermatitis skin and are not increased in lesional skin

**Figure 3:**
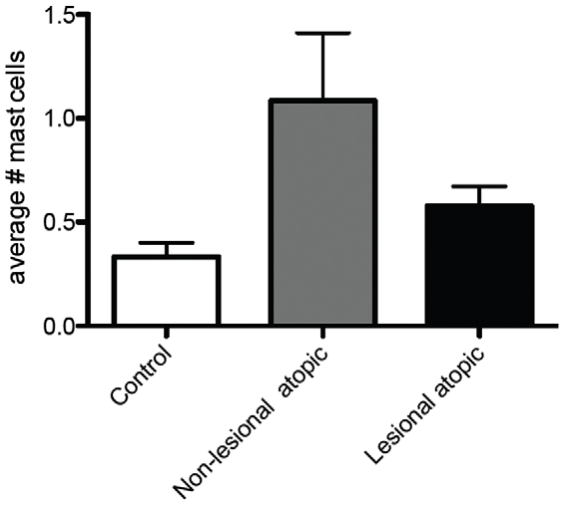
Mast cells in controls and atopic dermatitis patients. A non-significant increase in mast cells was seen in biopsies of non-lesional skin in atopic patients. There was no increase in mast cells in lesional skin.

Because mast cells are found in the skin in association with nerves, under both normal and pathological conditions [19], [20], and previous reports have shown mast cells are increased in lesional skin compared to healthy controls [21], [22], we stained biopsies with toluidine blue. A non-significant trend toward more mast cells was seen in non-lesional atopic dermatitis skin, but not in lesional skin (Figure 3).

### Nerves are increased in the epidermis of a transgenic mouse that expresses IL-5 in basal keratinocytes

**Figure 4:**
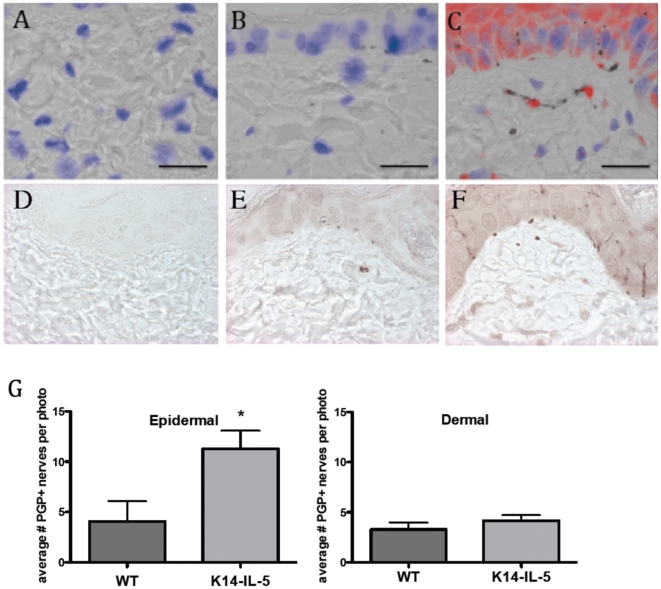
Keratin 14-Interleukin 5 mice have eosinophil major basic protein (MBP) primarily in the epidermis, and have more nerves in epidermis and basement membrane zone than wild-type controls, but similar numbers in the dermis. 5 um skin sections on slides were stained using anti-MBP (red, in top three sections) and anti-PGP9.5 to visualize nerves (brown, in bottom three sections). Photographs of each entire section were taken, and random numbers assigned to each for quantification of nerves by an observer blinded to the genotype. (A and D) IgG negative control, (B) anti-MBP staining of wild type mouse,(C) anti-MBP staining of K14-IL-5 skin section. MBP staining is largely seen in the epidermis, which is where the IL-5 is expressed. (E) PGP9.5 stained wild type mouse, (F) PGP9.5 stained K14-IL-5 skin section. Note the extensive linear nerves from the basement membrane zone through many layers of the epidermis. (G) Quantification of nerves shows an increase in the epidermis (where IL-5 is expressed and eosinophil major basic protein is seen) but not in the dermis. Scale bars = 50 µM.

NJ.692 mice were generated to constitutively express IL-5 under the control of keratin 14 regulatory elements in basal keratinocytes. The mice have mild peripheral blood eosinophilia with greater than 30-fold increased numbers of eosinophils in the skin. Some mice develop spontaneous skin lesions and clinically apparent inflammation. These animals also have deposition of eosinophil granule proteins in the skin, predominantly in the epidermis, which can be detected using anti-MBP antibodies (figure 4C).

We examined skin sections from these mice for the number of nerves and found that they have more nerves in the epidermis and basement membrane zone than wild-type controls, but similar numbers of nerves in the dermis (Figure 4). Thus the increase in nerves was seen in the same regions where IL-5 was expressed and where eosinophils were located.

### Eosinophils increase sensory neuron branching *in vitro*

**Figure 5:**
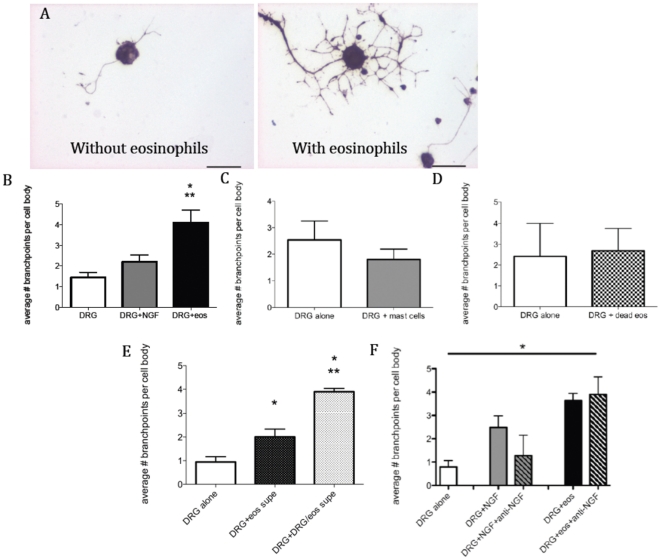
Eosinophils increase branching of dorsal root ganglion (DRG) neurites. (A) Mouse DRG neurons were isolated and co-cultured with eosinophils for 24 hours. Cells were fixed and stained with anti-PGP9.5 to visualize neurons. DRG neurons cultured with eosinophils (right) showed dramatic increases in neurite branching, compared to DRG neurons cultured alone (left). (B) Quantification of number of branchpoints per cell body, n = 7. * denotes significantly different from DRG alone, ** denotes significantly different from DRG+NGF. (C) Mast cells were isolated from the peritonea of wild-type mice and added to DRG neuron cultures for 24 hours. Slides were stained and quantified as above. n = 4. (D) Eosinophils were resuspended in sterile deionized water and freeze-thawed twice, resuspended in culture medium and added to DRG neuron cultures for 24 hours. n = 2. (E) Culture medium from eosinophils cultured alone or with DRG for 24 hours was applied to new DRG cultures for 24 hours. n = 4. (F) DRG neurons were plated and incubated alone (white bar), with 40 ng/ml NGF (gray solid bar), with NGF and 20 ug/ml anti-NGF (gray hatched bar), with eosinophils (black bar), or with eosinophils and 20 ug/ml anti-NGF (black hatched bar) for 24 hours. n = 2. * denotes significant one-way ANOVA across all groups.

The cell bodies of the sensory neurons that innervate the skin are located in the dorsal root ganglia (DRG), and these were dissected from wild-type mice, enzymatically dissociated and plated on Matrigel. Blood was taken from NJ1638 mice, in which the IL-5 gene is expressed under the control of the CD3δ promoter, leading to high levels of circulating eosinophils [23]. Eosinophils were isolated using density centrifugation and fluorescence-activated cell sorting (FACS) of unstained cells based on size and granularity. Eosinophils were added to DRG cell cultures for twenty-four hours. The cultures were then fixed, stained with PGP9.5, and the number of cell bodies, length of neurites, number of neurites per cell body, and number of branchpoints per neurite were quantified by an observer who was blinded to the experimental condition. Neurons grown with eosinophils had dramatically increased neurite branching, compared to neurons grown alone (Figure 5 A, B). The number of cell bodies, number of neurites per cell body and the length of the longest neurite were not significantly different between neurons alone or neurons with eosinophils (data not shown). There was also no difference in DRG cell survival with or without eosinophils (data not shown).

### Mast cells do not increase neuron branching *in vitro*

Since mast cells were localized around nerves in human skin biopsies, we isolated mast cells from the peritonea of wild-type mice and added them, in the same numbers as eosinophils, to the neuron cultures for twenty-four hours. Mast cells did not increase neurite branching (Figure 5C).

### Eosinophils must be alive to increase neuron branching, but physical contact is not required

In order to determine whether the eosinophils needed to be alive to have this effect on neurite branching, we freeze-fractured eosinophils and added them to neuronal cultures for twenty-four hours. Eosinophils killed in this way did not increase neurite branching (Figure 5D). Adding culture medium from eosinophils plated on Matrigel for twenty-four hours to DRG cultures increased neuron branching, indicating that the morphological changes do not require cell contact, and adding culture medium from co-cultures of DRG neurons with eosinophils to fresh DRG cultures further increased the amount of neurite branching (Figure 5E).

### Blocking nerve growth factor (NGF) does not inhibit eosinophil-induced neurite branching

Adding exogenous murine NGF (40 ng/ml) to DRG cultures increased neurite branching, and this effect was blocked by an antibody to NGF (20 ug/ml) (Figure 5F). However, adding the same concentration of anti-NGF to DRG cultures co-cultured with eosinophils (Figure 5F) or treated with eosinophil culture medium (data not shown) did not prevent the increase in neurite branching. Thus NGF production by eosinophils was not responsible for the effect on nerve branching.

### Neurons from dorsal root ganglia produce eotaxin-1, ICAM-1 and VCAM-1

**Figure 6:**
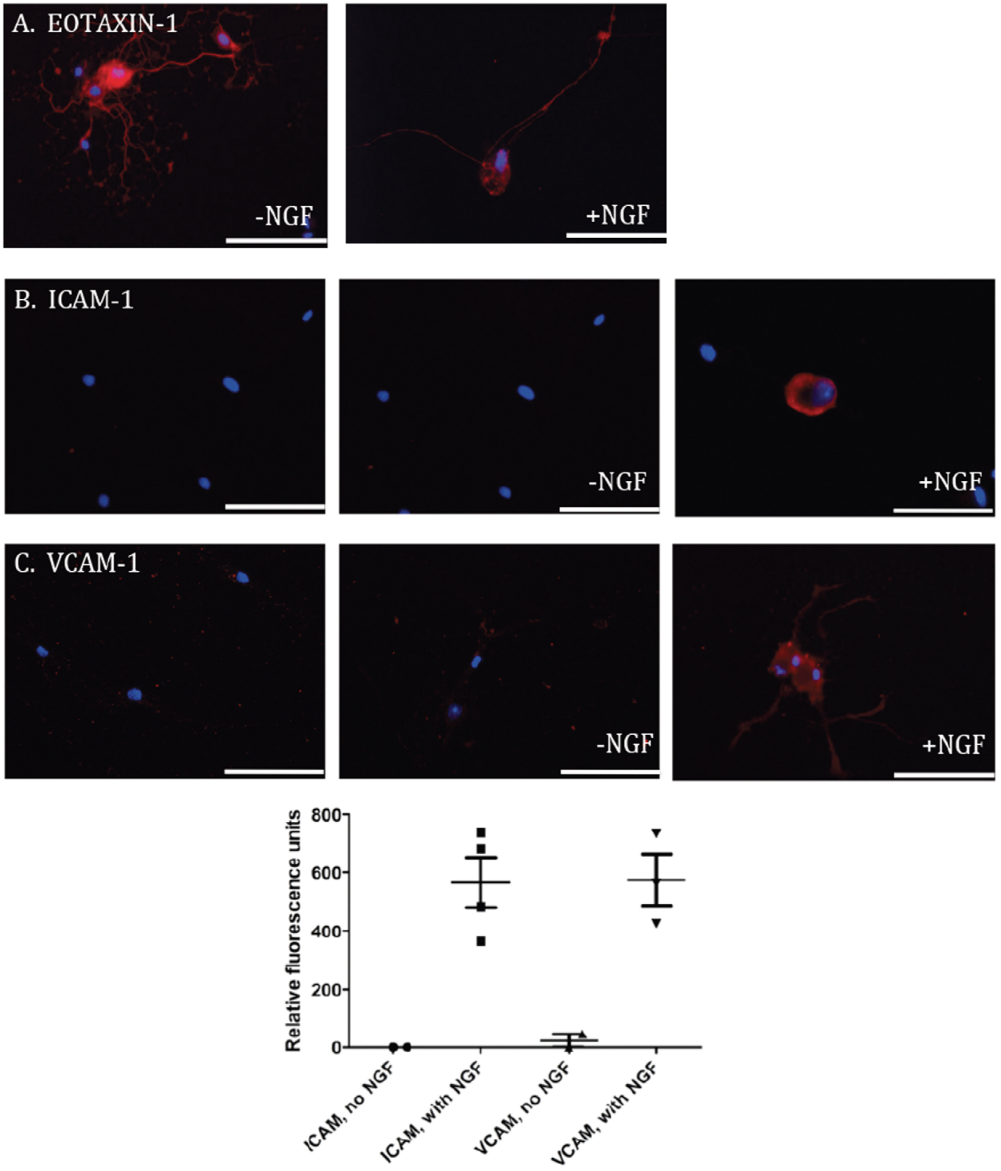
DRG neurons produce eotaxin-1, ICAM-1, and VCAM-1. (A) DRG neurons were isolated and cultured alone for 24 hours, then fixed and stained with anti-eotaxin-1 (red). Cell nuclei were counterstained using DAPI (blue). Two representative photomicrographs are shown. Scale bars = 50 um. (B–C) DRG neurons were cultured alone or with 40 ng/ml NGF for 24 hours, then fixed and stained with anti-ICAM-1 (B) or anti-VCAM-1 (C). Left, no primary with secondary negative control; center, DRG stained with antibody in cultures without exogenous NGF; right, DRG stained with antibody in cultures with NGF added. GRAPH: Quantification of relative fluorescence units, compared to background and to negative controls, was performed using Metamorph.

DRG neurons were cultured alone for twenty-four hours and stained for eotaxin-1, a chemokine that acts through the CCR3 chemokine receptor on eosinophils [24], [25]. Neurons synthesized eotaxin-1, both in the presence and absence of exogenous NGF (Figure 6A). In addition, neurons also produced the adhesion molecules ICAM-1 and VCAM-1, but only when exogenous NGF was added to the cultures (Figure 6B, C). This indicates a regulated expression of adhesion molecules that could be affected by the presence of eosinophils or other cells that express NGF [9], [26].

## Discussion

Our data demonstrate for the first time that eosinophils can dramatically increase sensory neuron branching, and that this effect is important in eosinophilic skin diseases, such as atopic dermatitis. We found that there were more nerves in lesional versus non-lesional atopic dermatitis, which correlated with the amount and location of eosinophil granule proteins. In transgenic mice that express IL-5 driven by a K14 promoter, both eosinophils and increased innervation were localized to the epidermis. Finally, eosinophils dramatically increased neurite branching in cultured dorsal root ganglion sensory neurons. The ability of eosinophils to promote neurite branching in sensory neurons in culture supports the role of eosinophils in the neural changes in atopic dermatitis. Sensory neuron expression of ICAM-1 and VCAM-1, as well as eotaxin-1, suggests active recruitment of eosinophils to the nerves, as we have previously demonstrated for parasympathetic neurons in the airways [13].

Our biopsies were taken from several places anatomically, and the control patients' biopsies were all taken from the inner arm (Table 1). While this might have affected the number of nerves, the regional variation in cutaneous nerve density would favor more nerves in the arm than in the leg [27], and we found the opposite when comparing atopic dermatitis biopsies to control biopsies. Although there was a general increase in cutaneous mast cells in our human skin biopsies, unlike the eosinophils cutaneous mast cells were not increased in the lesional skin, where nerves were increased. This finding, along with the failure of mast cells to promote nerve branching in vitro, suggests that eosinophils are more likely responsible.

In human and mouse skin, we did not identify which types of nerves were increased in number, although the locations of these nerves in the papillary dermis and along the basement membrane zone would suggest sensory origins. However, our culture experiments specifically examined the branching of sensory neurons with cell bodies in the dorsal root ganglia, which include the itch-specific C-type neurons [2], [28], [29].

Itch-specific C-type neurons are reported to have more highly branching terminals, enabling them to innervate exceptionally large regions of tissue [29]. It is possible that eosinophils are not simply stimulating neurite growth but are causing a shift in the phenotype of the affected neurons. A precedent for this type of shift lies in *hyperalgesia* and *allodynia*, in which changes in neurotransmitter release, ion channel regulation and threshold for stimulation lead to perception of non-painful stimuli as pain [30], [31].

We showed that NGF was not the necessary factor for eosinophil-induced neurite branching using a blocking antibody that has less than 1% cross-reactivity for BDNF and neurotrophins 3 and 4 (NT-3, NT-4). However, eosinophils synthesize other neurotrophins, including BDNF and NT-3 [18], [32], as well as interleukins and other mediators that could be responsible for nerve growth. Thus there may be considerable redundancy in this system, as well as the possibility that other, non-neurotrophin eosinophil products, are involved. For example, major basic protein, the principal constitutent of the eosinophil granule, interacts with neurons on several levels, including blocking M2 muscarinic receptors on the nerves [15], increasing sensory nerve excitability [33], and activating p38 MAP kinase [34]. The effects of major basic protein, as well as other eosinophil granule proteins and non-granule products, is not known.

Our findings are also important for other eosinophilic skin diseases, several of which induce itch as a predominant symptom. Itchy papular eruptions have been described in the hypereosinophilic syndrome (HES) and can be successfully treated with ultraviolet (UV) therapy [35]. In prurigo nodularis, a disease defined solely by its itchiness, large deposits of eosinophil cationic protein (ECP) and EDN were detected by immunofluorescence. Intact eosinophils were often located near nerves, and there was an increase in the number of nerves in areas with many eosinophils [36]. Finally, a disease called equine sweet itch results from sensitization to the bite of the midge and subsequent “challenge” or further bites. Eosinophils are recruited by eotaxin and MCP-1, which are expressed in the skin of sensitized horses, and horses scratch, rub and bite the lesions, which results in skin thickening and hair loss [37].

Our report demonstrates the role of eosinophils in nerve branching *in vivo* and *in vitro*, and strongly suggests that this is relevant to human skin diseases, including atopic dermatitis, in which eosinophils and nerves are found together. The ability of sensory neurons to express adhesion molecules and chemokines that interact with eosinophils suggests potential drug targets in eosinophil-related skin diseases.

## Methods

### Human Skin Biopsies

Human studies were approved by the Institutional Review Board of Oregon Health & Science University (IRB approval #2568), and all patients gave written informed consent. Healthy subjects and atopic dermatitis patients were interviewed for their personal and family history of atopy and then examined by a dermatologist to determine extent of disease. A self-reported history of atopy was required for inclusion as an atopic dermatitis patient, and all patients had a previous diagnosis of atopic dermatitis by dermatologists at OHSU. Four millimeter punch biopsies were removed from areas of normal-appearing (non-lesional) or active (lesional) disease. Biopsies of normal skin from healthy subjects were taken from the inner arm or behind the knee. All biopsies were fixed in 10% neutral buffered formalin and embedded in paraffin.

### Human Skin Immunocytochemistry

5 um paraffin sections were cut onto slides, and these were rehydrated through xylenes and sequential ethanol dilutions. Antigen unmasking solution was used according to the protocol, [Vector], and 3% hydrogen peroxide in methanol was applied for ten minutes, to quench endogenous peroxidase activity. Sections were blocked with 10% normal goat serum, and mouse anti-human protein gene product 9.5 (PGP9.5) [Serotec] was applied overnight at 4°C. Slides were rinsed in PBS and biotinylated goat anti-mouse IgG was applied for 30 minutes at room temperature. The Vectastain ABC kit and chromogenic substrates were used to visualize positive antibody staining. Slides were rinsed in tap water, then dehydrated through ethanol dilutions and xylenes. Slides were mounted using Cytoseal CrystalMount and dried overnight. Likewise, human skin biopsies stained for eosinophil peroxidase (EPO) were blocked and incubated with antibody at 4°C overnight, then treated identically as PGP9.5-stained slides. Sections stained for both EPO and PGP9.5 were treated with the avidin-biotin blocking kit from Vector to prevent non-specific signal. Sections used for H&E or mast cell identification were deparaffinized and rehydrated as above and then stained using hematoxylin and eosin or toluidine blue.

### Semi-quantitative Analysis of Immunostaining of Human Skin

40× or 60× photomicrographs were taken of the entire region of each skin biopsy. Measurements were taken of papillary dermis, reticular dermis, hypodermis, and epidermal-dermal zone; however, only measurements of papillary dermis and epidermis/basement membrane are reported. Nerve length was calculated by calibrating each photograph to the objective with which it was taken and then drawing a straight line between the two furthest points of a PGP 9.5-positive nerve, using Metamorph software. Nerve number was counted manually. Data were reported as average number of nerves per photo, and average length of nerve per photo. Mast cells were counted in double-stained 60× photographs, and proximity to nerves measured using Metamorph software. Fifteen microns, or approximately half a mast cell's diameter, was determined to be the limit of “association” between a mast cell and nerve.

### Animals

Female wild-type C57BL/6 mice, 6–8 weeks of age, were purchased from Jackson Laboratories. NJ.1638 IL-5 transgenic mice, in which the full murine IL-5 sequence, with every intron and 1.2 kb of 3′-flanking sequences, but none of the upstream known regulatory elements, is regulated by the promoter and tissue-specific enhancer for CD3δ, overexpresses IL-5 in T cells. These mice were generated, bred and genotyped as previously described (67). NJ.692 mice (K14/IL5 transgenic mice) were generated by creating a plasmid construct containing the 1.9 kb K14 promoter (provided by Dr. Elaine Fuchs [38] fused to the IL-5 gene. The plasmid insert was injected into mouse embryos following excision from the plasmid vector. Mice were bred with C57Bl/6J mice (Jackson Laboratories) and maintained using either Southern analysis of BamHI digested tail DNA blotted to radioactively labelled IL-5 cDNA or PCR reactions using primers specific for the IL-5 injected DNA. PCR primers were (K14) 5′ TTG GCG CTA GCC TGT GGG 3′ and (IL5 beta 2) 5′ CAG CCT ACC CTA CAT AGC AAG TTT G 3′. All animal experiments were approved by the Institutional Animal Care and Use Committee of Oregon Health & Science University (approval # B11249), and were performed in accordance with National Institutes of Health and Mayo Foundation institutional guidelines.

### Mouse skin immunocytochemistry

Skin sections from mice were fixed in 4% formaldehyde and embedded in paraffin. Five micron sections were immunostained in the same manner as human skin, using rabbit anti-mammal PGP9.5 and biotinylated goat anti-rabbit IgG. Sequential samples were double-stained with PGP9.5 and major basic protein (MBP) antibodies generated in the Lee Laboratory. Rat anti-mouse MBP was applied overnight at 4°C, and secondary antibody, conjugated to the fluorophore Alexa Fluor-555, was applied for 2 hours at 37°C. Slides were rinsed in PBS, and mounted using Vectastain with DAPI, to stain cell nuclei.

### Semi-quantitative analysis of mouse skin immunohistochemistry

60× photographs of entire mouse skin biopsies were taken. Slides were de-identified using an algorithm assigning random numbers to each photograph, and measurements were taken of PGP-positive spots in the papillary dermis, reticular dermis, and epidermal-dermal zone of each blinded photograph by an investigator blinded to the condition of the biopsy. Nerve length was calculated by calibrating each photograph to the 60× objective and then drawing a straight line between the two furthest points of a PGP 9.5-positive nerve, using Metamorph software. Nerve number was counted manually. Data from each photograph were averaged to determine the mean number of nerves per photo, sum of nerve lengths per photo, and average length of nerve per photo. Each photograph was de-coded after all measurements and calculations were completed. Quantification of nerves per area or nerves per length of basement membrane did not give different results from number of nerves per photo.

### Isolation of Dorsal Root Ganglia (DRG)

Dorsal root ganglia were isolated according to modifications of previous published protocols [39], [40]. Cervical, thoracic and lumbar DRG were dissected from the spines of wild-type C57BL/6 mice and incubated in 0.05% collagenase at 37°C for four hours, centrifuged at 300×g for 10 minutes at room temperature, and resuspended in 3 ml 1.25% Trypsin-EDTA for 15 minutes at 37°C. Cells were centrifuged again and resuspended in DMEM, 10% FBS, penicillin-streptomycin, and pre-plated. The next day, non-adherent cells were centrifuged and resuspended in C2 nerve growth medium that contained penicillin-streptomycin, at a concentration of 5×10^5^ cells/ml. Nerves were plated on Matrigel-coated chamber slides. Murine NGF was added to some cultures at a concentration of 40 ng/ml. Sheep anti-NGF was added for a final concentration of 20 ug/ml. At the end of each experiment, cells were fixed in 4% paraformaldehyde for 5 minutes.

### Isolation of murine blood eosinophils

Diluted blood from NJ.1638 (IL-5 transgenic) mice was layered over 15 mL sterile Percoll (density 1.084 g/mL) and centrifuged at 2000×g for 45 minutes. The white layer at the interface, which contained the granulocytes, was collected, washed and centrifuged at 300×g for 15 minutes at 4°C. Red cells were lysed and the eosinophils were isolated by size and granularity using fluorescence-activated cell sorting (FACS). Trypan blue exclusion was used to determine viability. Percent purity was determined by Hemacolor assay on cytospin slides. After FACS staining, percent purity of eosinophils was determined by Hemacolor staining and differential cell counts under the 20× objective of a bright field microscope. The purity of eosinophils after sorting was 91%. Cells were resuspended in C2 nerve medium at the indicated concentrations and added to cultures of DRG neurons for 24 hours.

### Isolation of murine peritoneal mast cells

Peritoneal mast cells were isolated according to the method of Jensen et al. [Jensen B, Swindle E, Iwaki S, Gilfillan A, Current Protocols in Immunology, 2006, Wiley Interscience]. A single mouse was killed and the peritoneum was lavaged with HBSS. Lavage fluid was centrifuged at 400×g for 5 minutes, red blood cells were lysed, and the cells were washed twice with HBSS. The pellet was resuspended in 70% isotonic Percoll solution, and cells were layered over filter-sterilized peritoneal mast cell (PMC) medium (DMEM, 5% FBS, 2 mM L-glutamine, 50 ug/ml gentamicin, 20 mM HEPES) and centrifuged at 580×g for 15 minutes at room temperature. The mast cell pellet was resuspended in PMC medium. Aliquots of cells were counted and stained with trypan blue to determine viability or toluidine blue to determine purity of mast cells. Finally, cells were adjusted to indicated concentrations, added to slides or wells and incubated at 35.5°C with 5% CO2.

### Dorsal Root Ganglion Immunocytochemistry

Slides with DRG were fixed in 4% paraformaldehyde for 5 minutes, rinsed with HBSS with calcium and magnesium, and stored in PBS until stained. Slides were blocked with 10% serum and stained with antibodies to PGP 9.5, ICAM-1, VCAM-1, or eotaxin-1 overnight at 4°C. After rinsing in PBS, a secondary antibody conjugated to Alexa Fluor-555, or biotin, was applied. Slides were rinsed and either mounted in Vector Mounting Medium with DAPI, to stain cell nuclei, or completed, using Vector Vectastain Avidin-Biotin Complex immunohistochemistry kit and mounted.

### Dorsal Root Ganglion Imaging and Quantification

Thirty 40× photographs were taken of each chamber after each treatment, stained with anti-PGP 9.5 antibody. Photographs were always taken beginning in the center of each well of a 4-well chamber slide, and then every subsequent field of view was photographed, in the same defined pattern for all slides. Using Metamorph, numbers of cell bodies, neurites, and branchpoints per cell body were counted manually. Each cell body in each photograph was quantified. In addition, neurite length was measured with the segmented line function, which allowed the neurite to be traced from end to end and measured according to the pixel per micron ratio for the 40× objective. The mean of each measurement (number of cell bodies, neurites, branchpoints and neurite length) for all cell bodies in thirty pictures was determined. Verification of this method was provided two ways: first, one group of photographs each from two experiments was quantified by a blinded observer, who knew neither the treatment groups nor the predicted outcome, and second, photographs were taken at random places in each well for one experiment, and a blinded observer then performed the measurements. Blinded observations were found to match unblinded observations.

### Statistical Analysis of Data

All data are expressed as mean +/− SEM. Comparisons between two groups were made using t-tests for unpaired data, while comparisons of nerves in lesional and non-lesional skin from the same human were made using paired t-tests. Multiple comparisons used one-way ANOVA, with a Tukey's post-test to evaluate differences between groups. A p value of less than 0.05 was considered statistically significant.
